# Squalene: More than a Step toward Sterols

**DOI:** 10.3390/antiox9080688

**Published:** 2020-08-02

**Authors:** Marco Micera, Alfonso Botto, Federica Geddo, Susanna Antoniotti, Cinzia Margherita Bertea, Renzo Levi, Maria Pia Gallo, Giulia Querio

**Affiliations:** 1 Department of Life Sciences and Systems Biology, University of Turin, 10123 Turin, Italy; marco.micera@unito.it (M.M.); federica.geddo@unito.it (F.G.); susanna.antoniotti@unito.it (S.A.); cinzia.bertea@unito.it (C.M.B.); renzo.levi@unito.it (R.L.); giulia.querio@unito.it (G.Q.); 2 Exenia Group S.r.l., 10064 Pinerolo (TO), Italy; info@exeniagroup.it

**Keywords:** squalene, oxidative stress, cardiovascular diseases, antioxidants, ROS, RNS

## Abstract

Squalene (SQ) is a natural triterpene widely distributed in nature. It is a metabolic intermediate of the sterol biosynthetic pathway and represents a possible target in different metabolic and oxidative stress-related disorders. Growing interest has been focused on SQ’s antioxidant properties, derived from its chemical structure. Strong evidence provided by ex vivo models underline its scavenging activity towards free radicals, whereas only a few studies have highlighted its effect in cellular models of oxidative stress. Given the role of unbalanced free radicals in both the onset and progression of several cardiovascular diseases, an in depth evaluation of SQ’s contribution to antioxidant defense mechanisms could represent a strategic approach in dealing with these pathological conditions. At present experimental results overall show a double-edged sword role of squalene in cardiovascular diseases and its function has to be better elucidated in order to establish intervention lines focused on its features. This review aims to summarize current knowledge about endogenous and exogenous sources of SQ and to point out the controversial role of SQ in cardiovascular physiology.

## 1. Introduction

Squalene (SQ) is a lipophilic biomolecule that belongs to the chemical class of triterpenes. It is formed by six isoprene units, so, it is a 30-carbon isoprenoid compound with six double bonds (C_30_H_50_). SQ is an odorless, colorless, liquid oil. The molecule was named squalene because it was initially isolated by Tsujimoto from shark (*Squalus milsukurii* and other squaloids) liver oil [1,2,3], but subsequent studies have also found this compound in several plant extracts [4].

**Figure 1:**
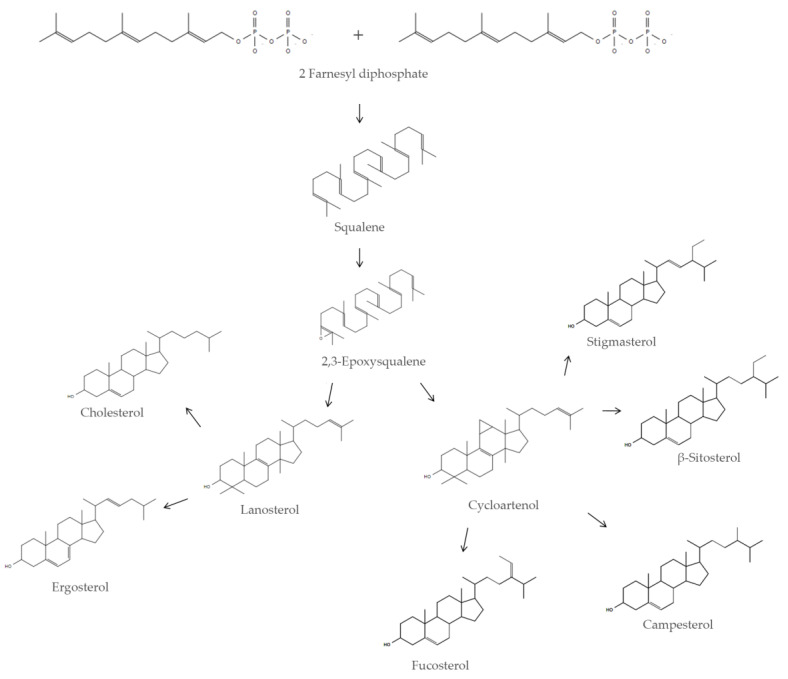
Biosynthetic pathway of animal and plant sterols. Squalene is synthesized from two units of farnesyl diphosphate by squalene synthase and then converted in 2,3-epoxysqualene, the precursor of animal and plant sterols. The animal sterols cholesterol and ergosterol originate from lanosterol, while the plant sterols, stigmasterol, β-sitosterol, campesterol and fucosterol, originate from cycloartenol.

In eukaryotic cells SQ is a metabolic intermediate in the sterol biosynthetic pathway [5]; squalene synthase, an enzyme associated with the membrane of the endoplasmic reticulum, catalyzes the biosynthesis of SQ in a two-step reaction starting from two units of farnesyl diphosphate [6]. SQ is the precursor of cholesterol in animal cells and the precursor of phytosterols in plant cells (Figure 1).

As an intermediate in sterol biosynthesis, SQ plays a crucial role in plant and animal organisms [1,7]. Several in vitro and in vivo studies have already underlined the antioxidant effects of SQ, probably due to its molecular properties directly linked to its activity towards free radicals [1,2,8]. In fact, due to its conformation, it can easily move through cellular and subcellular membranes, allowing rapid distribution in every cell compartment [9]. In addition, it acts as scavenger and quencher against some free radicals. In particular, SQ breaks lipid peroxidation chains both in the initial phase and in the propagation phase [8].

However, SQ’s role in the prevention or treatment of oxidative stress-related cardiovascular diseases (CVDs) is still controversial and further studies are needed to investigate the correlation between high plasma SQ concentrations and CVDs.

This work focuses on current knowledge about SQ roles in humans, its endogenous and exogenous sources, and its beneficial and negative impact on health, with particular attention to metabolic and cardiovascular disorders.

## 2. Sources of Squalene

### 2.1. Endogenous Sources of Squalene

In human body, squalene is present in multiple organs and tissues. As expected, the liver is the main player of its biosynthesis and this organ represents the primary way through which SQ enters the systemic circulation. Animal studies demonstrate that interference in cholesterol biosynthesis in liver could influence squalene concentration both at the local (in hepatocytes) and systemic levels [10].

SQ is present in plasma embedded into lipoproteins: its concentration is particularly high in very low density lipoproteins (VLDL), but significant concentrations of SQ are also found in low density lipoproteins (LDL) and high density lipoproteins (HDL), and its amount seems to be directly correlated with triglyceride abundance in these components [11,12].

Because of SQ hydrophobic nature, it could be found in a concentration of 275 µg/g in adipocytes [13]. Squalene constitutes about 12% of the human skin surface lipids [14], probably because, compared with other mammals, human skin is not so hairy, and therefore it needs more strategies to counteract the photo-oxidative action of UV rays [15]. Moreover, several research studies have pointed out SQ high concentrations (about 475 µg/g) in epithelial tissue, suggesting a possible in loco synthesis of this compound, where sebaceous glands figure as probable sources of SQ [4,11,13].

### 2.2. Exogenous Sources of Squalene

Recent studies have highlighted exogenous natural or synthetic sources of squalene. Indeed, SQ can be introduced through food products, food supplements and drugs [2]. As previously mentioned, SQ was first isolated from shark liver oil in which its concentration reaches nearly 40% of the total oil [2,11,13]. A shark’s liver can constitute up to 25% of its body mass, and the role of squalene in the liver is known to contribute together with diacyl glycerol esters to the control of animal flotation [16]. Several works point out and support its dermatological use [15]. Fortunately, today cosmetic industry is strictly regulated by laws which protect these animal species and SQ has to be isolated from other natural or synthetic sources.

In this scenario plants, where it is a precursor of phytosterols and other secondary plant metabolites, figure as alternative natural sources of squalene. SQ is highly concentrated in the unsaponifiable part of several plant oils. Furthermore, several studies demonstrate that plant levels of squalene are strictly correlated to the plant source, the location and period of plant growth and the extraction method used to separate SQ from other metabolites [11]. Considering all these factors, it could be necessary to use high quantities of raw materials to extract SQ from plant rather than animal sources. For these reasons several methods have been applied in order to reach higher concentrations of SQ in plant extracts. In addition to traditional mechanical and chemical extraction, using ethanol [17], new approaches have been developed with successful results, such as supercritical CO_2_ extraction [17,18,19].

The most relevant plant sources of SQ are oils extracted from amaranth, olive, rice, wheat germ, grape seed, peanut and soybean [4,11,20]. As previously mentioned the abundance of SQ in the extract is influenced by several factors, but promising results obtained in this research field offer positive data on the possible use of plant-derived SQ in pharmaceutical and cosmetic sectors and in the nutraceutical field.

Regarding exogenous SQ fate in the digestive tract, animal and human studies have already tested the bioavailability of dietary SQ, underlining that nearly 60% of the ingested compound is absorbed. Non-absorbed SQ is in part excreted with feces and the rest probably metabolized by the gut microbiota, however further studies are needed to elucidate this pathway [21,22].

## 3. ROS/RNS and Squalene

### 3.1. ROS/RNS and Squalene

Oxidative stress leading to lipid peroxidation is triggered by different oxidizing molecules and, among them, O_2_^●^ is the first reactive oxygen species (ROS) involved in the damage of cell membranes. The presence of SQ seems to reduce this radical by releasing an electron, through reaction (1), at the moment observed only in ex vivo models and not yet demonstrated in cellular systems:

SQ + O_2_^●^→ HOO-SQ (1)

The stabilization of the radical occurs through the formation of a squalene hydroperoxide (HOO-SQ) and the electronic rearrangement of SQ by resonance, a typical phenomenon related to molecules containing a number of double bonds [23]. This reaction leads to the formation of different isomers, which are specifically: 2-OOH-SQ, 3-OOH-SQ, 6-OOH-SQ, 7-OOH-SQ, 10-OOH-SQ, 11-OOH-SQ. In particular, the study by Shimizu et al. [14], examined this behavior by oxidizing SQ with 3-(1,4-epidioxy-4-methyl-1,4-dihydro-1-naphytyl)propionic acid (EP), a compound used to release O_2_ by thermal degradation. SQ oxidation occurs in a dose dependent fashion and the different isomers are produced in the same percentages. During this reaction O_2_^●^ acts as an oxidizing agent and reacts with a double bond to form hydroperoxides through the ene-reaction [24].

The rate of this reaction is expressed through a kinetic constant: the quenching constant (Kq). Krasnovsky and colleagues calculated this value as the sum of three other kinetic constants [25]: K_ph_: expresses physical quenching; K_1_: expresses kinetics reaction occurring between an oxidizing agent and an allylic hydrogen atom of the reducing agent, which is part of a methylene group between C=C and C–C; K_2_: expresses kinetics reaction occurring between an oxidizing agent and an allylic hydrogen atom of the reducing agent, which is part of a methylene group between C=C and C=C. Since SQ does not have conjugated double bonds, this value is equal to 0.

Squalene Kq (Kq_SQ_) has been measured by Kohno et al. [8], in an environment saturated with butanol at 35 °C. Kq_SQ_ (2.66 × 10^6^ M^−1^ s^−1^) is greater than the Kq of some membrane lipids, such as ethyl linolenate, eicosapentaenoic acid ethyl ester and docosahexaenoic acid ethyl ester. Since the Kq of saturated and monounsaturated fatty acids is lower than that of polyunsaturated acids [25], and Kq_SQ_ shows the highest value among membrane polyunsaturated acids, the stabilization reaction will take place faster in presence of SQ rather than other membrane lipids. Therefore, SQ oxidation occurs earlier than other lipids, blocking the peroxidative reactions and thus protecting cell membranes. Furthermore, Kq_SQ_ has a lower value than Kq of α-Tocopherol, but similar to the Kq of dibutylhydroxytoluene (BHT) molecules, widely used as antioxidants in food industry.

Moreover, SQ is inserted in cell membrane and contributes to its stabilization [26,27]. In particular, in the plasma membrane, due to its hydrophobicity, it is oriented parallel to the plane of symmetry of the lipid bilayer, embedded between fatty acids terminal methyl groups of the two layers. The strategic position of SQ forms an intermediate barrier between the two phospholipidic layers and allows the molecule to act as a quencher that blocks uncontrolled electron flows. The role of SQ in stabilizing cell membranes underlines its important role also in cardiac cell membrane reconstitution and in the regulation of membrane proteins and ion movement [26].

### 3.2. Oxidative Stress in Skin and Squalene

Antioxidant properties of SQ were primarily studied in epithelial tissue. In fact, the molecule is highly abundant in skin, that is the largest tissue exposed to different environmental stressors leading to oxidative stress, such as pollutants, photo-oxidation and UV-light. In particular, Aioi and colleagues show the scavenging activity of SQ on superoxide anion formation in keratinocytes exposed to oxidative stressors [28], suggesting a protective role of the molecule that acts in combination to superoxide dismutase.

### 3.3. Cardiovascular Antioxidant Systems and Squalene

Recent studies, aiming to evaluate the effect of SQ on antioxidant enzyme levels and activity in cardiovascular and hepatic tissues, showed a positive correlation between SQ and glutathione and non-glutathione-dependent enzymatic defenses. In fact, even if the first antioxidant activity of SQ is linked to lipid peroxidation blockade, as already described, growing evidence is emerging on its involvement in the regulation of the expression and activation of glutathione peroxidase (GPx), catalase (CAT), superoxide dismutase (SOD) and glutathione S-transferase (GST) [29,30]. SQ prevents the alterations of SOD and CAT by detoxifying the cytosolic environment. Moreover, it acts reconstituting GSH and safeguarding the enzymatic activity of GPx. Furthermore, the GSSG/GSH ratio, which is considered as a marker of oxidative stress status, decreases after SQ treatment [30].

The positive influence of SQ on antioxidant enzymes has been demonstrated in stress situations, in particular, in animal models of isoproterenol-induced myocardial infarction [29]. Summarizing these results, it could be reasonable thinking the possible use of SQ in maintaining antioxidant enzyme activity in the heart and vascular system.

Table 1 summarizes different works in which the antioxidant properties of SQ were evaluated in cardiovascular system and in liver.

## 4. Oxidative Stress Associated CVDs and Squalene

The endothelium lining the lumen of blood vessels is pivotal in maintaining vascular function and its regulation has an important redox component. In fact, physiological ROS levels contribute to vasodilatation, angiogenesis, vascular remodeling and maintenance of lower blood pressure [34]. The main source of ROS in the vasculature is the NADPH oxidase (NOX). NOX-derived ROS act as essential second messenger molecules contributing to the regulation of normal cell function [35]. NOX4 and NOX2 isoforms are the major ROS producers in vascular smooth muscle cells and endothelial cells. In particular, H_2_O_2_ produced by NOX4 activates eNOS leading to the generation of NO, which exerts vasoprotective effects on the endothelium keeping blood vessels dilated and controlling blood pressure [36,37]. Moreover, vascular endothelial growth factor (VEGF), a physiological regulator of angiogenesis, induces endothelial ROS production through NOX4 and NOX2 activation and promotes endothelial cell proliferation, migration and survival [38]. In addition, normal levels of ROS are critical for the physiological response of vascular smooth muscle cells. In particular NOX4-induced H_2_O_2_ promotes cell differentiation and the phenotypic switch from a proliferative state to a contractile state, regulating the vascular tone [39].

Moreover, NOX4 is expressed not only in endothelial and vascular smooth muscle cells, but also in cardiomyocytes, in which it regulates autophagy, leading to its steady activation, and cytoprotective and antioxidant pathways [40].

Adverse cardiocirculatory events induced by oxidative stress are primarily linked to endothelial dysfunction [41,42,43,44], characterized by a complex multifactorial pathway, including eNOS uncoupling and increased concentration of inflammatory cytokines, both exacerbating the damaging effect induced by ROS/RNS boost [41]. Among risk factors that contribute to the development of endothelial dysfunction, some are classified as non-modifiable factors, as aging and family history of CVDs, other are classified as modifiable risk factors and are related to lifestyle [41].

The following paragraphs describe the role of SQ in different oxidative stress associated CVDs, that are strictly linked to endothelial dysfunction development, and underline the possible use of SQ as a natural supplement to support standard drug therapy.

### 4.1. Hypercholesterolemia

Hypercholesterolemia is a metabolic disorder characterized by high levels of circulating low density lipoproteins (LDL) and related adverse effects. According to clinical health guidelines, reference values are: total cholesterol < 200 mg/dL, LDL < 100 mg/dL and HDL > 50 mg/dL; higher circulating values of total cholesterol and LDL have to be considered as hypercholesterolemic conditions [45].

Presence of high levels of LDL and ROS can contribute to the development of atherosclerosis: oxidized LDL (oxLDL) are taken up by macrophages that become foam cells; macrophages also produce cytokines that recruit T cells that are involved in amplifying the inflammation. Endothelial cells produce cytokines that stimulate smooth muscle cell proliferation and migration; foam cells and smooth muscle cells form a lipid core surrounded by fibrotic tissue, the atherosclerotic plaque, highly susceptible to rupture and thrombus formation [42]. Given the high instability of the plaque and the possible negative effects derived from its rupture, it is important to interfere with both its onset and progression with hypocholesterolemic agents.

SQ, as a precursor of cholesterol, could be reasonably considered as an exacerbating factor for hypercholesterolemia. Despite that, Khor et al. demonstrated that addition of SQ to a high fat diet in hamsters does not affect levels of LDL, HDL and triglycerides and that the increased cholesterol ester accumulation in SQ fed hamster liver could account for the hypocholesterolemic effects of the supplemented chow [46,47]. Furthermore, Kritchevsky and colleagues demonstrated that a SQ based diet for 7 weeks in rabbits does not induce atheroma formation neither is directly involved in atherosclerosis development [46,48]. Other studies underline sex-related responses to SQ supplementation in ApolipoproteinE (ApoE^-/-^) KO mice, a useful animal model for atherosclerosis: males show a reduction of atherosclerotic lesion area independently from plasmatic lipid panel, while females do not show a reduction of atherosclerotic lesion area, but have significantly reduced plasma levels of cholesterol and triglycerides [49]. Other studies pointed out the molecular mechanism involved in SQ effects, some of them highlighting the activation of liver X receptors (LXRs), nuclear receptors engaged in homeostasis of cholesterol, fatty acids and glucose. In particular, a recent study shows that SQ acts as a selective modulator of LXRs in macrophages, inhibiting cholesterol accumulation and promoting its efflux; moreover, in hepatocytes SQ activates PPARα, thus upregulating the expression of genes related to fatty acid uptake, fatty acid oxidation, ketogenesis and reverse cholesterol transport metabolism. Based on these considerations, SQ represents a natural compound that can be used in dyslipidemic patients without negative side effects induced by standard administered drugs [50,51].

Granados-Principal and colleagues also studied SQ role in atherosclerotic plaque formation: they observed a dose-dependent reduction of surface expression of the oxLDL receptor CD36, in monocytes and macrophages and a consequent reduction in oxLDL uptake; furthermore, SQ exerted antiproliferative effect on monocytes and macrophages, thus contributing in atherosclerosis decline [52].

SQ also seems to be directly involved in cholesterol synthesis break, through a negative feedback mechanism: SQ alone or in combination with hypocholesterolemic drugs, such as statins, promotes both the down-regulation of HMG-CoA reductase, a key enzyme in cholesterol biosynthesis, and the cholesterol and bile acid excretion [53,54].

### 4.2. Hypertension

Hypertension is a complex, multifactorial, and multisystem disorder. Several studies have shown that endothelial dysfunction is an early and common trigger of hypertension and it is characterized by an impairment of the physiological pathways controlling vascular tone, primarily the NO pathway. Burst of ROS in dysfunctional endothelial cells lead to the irreversible production of peroxynitrite (ONOO^−^); ONOO^−^ can rapidly diffuse trough the cell and oxidize proteins, inducing eNOS uncoupling and thus decreasing NO production [55]. Furthermore, high ROS levels enhance angiotensin II signaling in resistance arteries and induce vascular smooth muscle cell hypertrophy [38].

New antihypertensive approaches based on nutraceutical properties of some food and plant extracts and, among them, squalene has shown positive effects in lowering blood pressure. Martirosyan and colleagues reported that SQ-rich amaranth oil reduces systolic blood pressure in a concentration dependent way [56]. Furthermore, Liu et al. showed that orally administered SQ for 4 weeks in rats diminishes both plasma lipids and blood pressure, probably through a reduction in circulating levels of leptin [46,57].

### 4.3. Hyperglycemia-Dependent Endothelial Dysfunction

Hyperglycemia is characterized by high fasting glucose levels in plasma, >200 mg/dL, due to genetic factors or wrong lifestyle habits. Chronic high glycemia causes enhancement of different molecules, in particular, polyol, advanced glycation end products, protein kinase C and exosamine, all involved in multiorgan damages. ROS rising, due to NOX enhanced activity and eNOS uncoupling, is strictly related to endothelial dysfunction associated to hyperglycemia [58]. Whereby dietary interventions in reducing glycemia are scant, hypoglycemic drugs are prescribed.

In recent years, many studies have suggested the use of plant extracts in order to lower plasma glucose and to prevent the development of ROS-induced endothelial dysfunction: among them, squalene is proposed as an antioxidant molecule capable of lowering both endothelial ROS and glycemia in hyperglycemic patients. At this regard, Liu and colleagues observed a reduction in plasma glucose in male Wistar rats fed with a high SQ diet in a time-dependent trend [57], while, Widyawati and collaborators highlight an hypoglycemic effect of a SQ-rich extract of *Sygyzium polyanthum*, mediated by a reduced glucose absorption in the gut and by an increased glucose uptake in the skeletal muscle [59]. Conversely, Valdes et al. showed no significant blood glucose reduction by SQ in streptozotocin-induced diabetic mice, pointing out the need of further investigation to define the hypoglycemic role of SQ [60].

## 5. Pathological Implications of Squalene

As a precursor of cholesterol biosynthesis, SQ both from endogenous or exogenous sources [61,62], the roles of SQ as a marker or a risk factor in the development of CVDs are essential. In order to define SQ implication in cardiovascular pathology, a few studies underline the possible strategic role of the molecule in coronary artery disease (CAD) [63] and in the development of visceral obesity and metabolic syndrome [64,65]. Moreover, emerging therapies for dyslipidemia are addressed to squalene synthase as a possible pharmacological target [21,66,67].

### 5.1. Coronary Artery Disease

Coronary artery disease (CAD) is a pathological condition characterized by atherosclerotic plaque formation in coronary vessels and it is strictly related to oxidative stress status. CAD is a multifactorial pathology in which both genetic and environmental factors contribute to its development. Studies underline different risk factors that are involved in the progression of this condition, among others, high LDL and low HDL cholesterol in plasma, hypertension, diabetes, smoking, sex and age. CAD could open to more severe conditions such as myocardial infarction and heart failure, so, it is important to stop the progression of the pathology lowering existing risk factors [68]. Rajaratnam and colleagues underline that in menopausal women high plasma concentration of squalene could represent a risk factor for CAD: they found higher plasma SQ related to serum cholesterol in women with cardiovascular predisposition to CAD than in controls. Moreover, elevated concentration of SQ were found in atherosclerotic plaques delivered by LDL [63]. The authors ascribed the high serum concentration of SQ to the decreased cholesterol synthesis resulting from hypercholesterolemic diet, and suggest SQ as a marker for CAD diagnosis [63,69,70,71].

### 5.2. Metabolic Syndrome

Metabolic syndrome is a multifactorial disease characterized by dyslipidemia, hypertension, diabetes and obesity [72,73]. Several interventions are needed to reverse this condition: life style changes are the first line modifications, and, to overcome critical situations, drug administration could be necessary.

In this scenario, abdominal adiposity and obesity are evident phenotypes of metabolic syndrome, when other latent symptoms are not yet manifested. Peltola and colleagues highlighted that high levels of serum SQ are linked to visceral obesity; as adipose tissue synthesizes and stores SQ, which is only in part converted to cholesterol, the authors suggest that SQ in adipose tissue could have detrimental effects in abdominal obesity, thus suggesting SQ as marker for metabolic syndrome [65]. According to these results, Lupattelli and collaborators demonstrated that in metabolic syndrome low synthesis of cholesterol and high absorption of SQ occur, thus explaining its high plasma concentrations [64]. Only a few data are available defining the critical role of SQ in metabolic syndrome and in the control of the resulting systemic oxidative stress status. They open essential questions about its direct or simply marker role in the onset of the pathological picture typical of metabolic syndrome.

### 5.3. Squalene Synthase Inhibitors

In order to assess a complete description of current knowledge about squalene and CVDs. it could be necessary also to define new advances in pharmacological therapies for dyslipidemia and the role of SQ in this pathological condition, strictly associated with the rise of circulating free radicals. Today most used drugs to treat dyslipidemia are statins, that block cholesterol biosynthesis inhibiting HMG-CoA reductase activity, fibrates and bile-acid sequestrants [27,61,66]. New frontiers are under investigation because these drugs have several adverse side effects when chronically administered.

For this reason, other enzymes in the cholesterol biosynthetic pathway are under investigation as putative pharmacological targets; among them, squalene synthase (SQS) represents a good candidate because it catalyzes one of the last reactions in cholesterol synthesis; indeed, its blockade does not interfere with the synthesis of other fundamental intermediates in the pathway, such as geranylgeranyl diphosphate or ubiquinone, and because only moderate side effect has been highlighted using this approach [67].

As a consequence of inhibition of SQS, low levels of endogenous squalene are reached and this may suggest a possible role of the molecule in the onset of hypercholesterolemia that have to be confirmed with further investigations [61,62,66].

## 6. Nutraceutical Properties of SQ

Nutraceuticals are defined as foods or ingredients or natural extracts that have a role in promoting health, thus preventing or treating some pathologies [74,75]. The previously described multiple beneficial effects of SQ support its potential nutraceutical properties and its possible use as food additive to prevent or treat some pathological conditions [74,75]. Toxicological studies show that SQ is well tolerated when consumed orally and that it has faster time of absorbance when compared to cholesterol [4,26].

Even if some nutraceutical properties of SQ have already been mentioned, recent animal studies underline in a more detailed manner antioxidant and cardioprotective effects of SQ- enriched diets. Indeed, SQ enriched diets show beneficial effects on mitochondrial energy status, lipid peroxidation and membrane stability. A number of research groups observed a stronger scavenging activity of SQ compared to vitamin E in elderly people [31]. Administration of SQ seems to have several positive effects on cardiovascular system, reducing atherosclerosis through induction of paraoxonase 1 (PON1) protective against the development of atherosclerosis [33], enhancing peripheral blood flow and improving central arterial elasticity [76]. Moreover, chemoprotective effects of SQ are currently under investigation [32,77,78,79,80,81].

## 7. Squalene: Healthy or Harmful in CVDs? Summary and Future Directions

The aim of this paragraph is to summarize both positive and negative effects of SQ in CVDs, as emerged from the previous sections, and to try to dissolve the vagueness around its pathophysiological role.

As pointed out in Table 2, all the results suggesting a role of SQ in the onset of CVDs arise from correlation studies, while a number of research articles reporting positive role of SQ, properly deepen the molecular mechanisms involved and this is in agreement with Ibrahim and colleagues [46]. Among them, activation of LXRs in macrophages [50], activation of PPARα in hepatocytes [51], reduction of CD36 expression in monocytes and macrophages [52] and downregulation of HMG-CoA reductase in the liver [53], strongly support the hypolipidemic role of SQ and its resulting anti-atherogenic effect. Based on these observations, future studies in SQ and CVDs should focus on cellular and molecular pathways directly affected by the triterpene, thus integrating clinical, cellular, molecular and biochemical analysis.

## 8. Conclusions

Squalene is a key metabolite in the sterol pathway and it is pivotal in regulating cellular and systemic physiology in eukaryotic organisms. Its role as a metabolic intermediate underlines its involvement in the regulation of different biochemical pathways, such as the regulation of sterol homeostasis (Figure 1). Despite the wide distribution of SQ in animal organisms, its direct role in regulating both pathologic and non-pathologic conditions is an important frontier of investigation.

In particular, its involvement in oxidative stress responses in human is still debated. Several studies indicate a dual role of the compound primarily linked to its chemical structure: the possible implication of SQ in antioxidant cellular response, and, in the other hand, the possible contribution of SQ in exacerbating detrimental effects of oxidative stress associated diseases.

This review focuses on the role of SQ in the development of oxidative stress associated CVDs. Treatment of most common CVDs, such as hypercholesterolemia, hypertension and hyperglycemia, with SQ enriched-diet shows promising results, underlining both quenching activity and enhancement of antioxidant systems induced by SQ against ROS/RNS burst [46]. Moreover, giving the emerging pivotal role of LXRs as a beneficial modulator of cardiovascular physiology [82], and the recent findings of SQ as an agonist of LXRs [50], future studies could be aimed at investigating the potential link between squalene and LXRs in cardiomyocytes and endothelial cells and in deepen the resulting intracellular pathway.

Even if chemical characterization and ex vivo results suggest a possible protective role of SQ against oxidative stress and free radicals, further human trials are needed to fully elucidate the cellular pathways activated by SQ in the regulation of cardiovascular ROS/RNS production.

## Figures and Tables

**Table 1:** Principal studies showing antioxidant effect of squalene in skin, liver and cardiovascular system.

Study	Model	Results
Aioi A. et al., 1995 [28]	Keratinocytes from shaved dorsal skin of rats, measurements of O_2_^−^ by reduction of equine ferricytochrome *c* after treatment with SQ and stressing agent	Inhibition of O_2_^−^ production by addiction of 100 mg/mL SQ
Buddhan S. et al., 2006 [31]	Young and old male Wistar albino rats fed with a 2% SQ supplemented diet for 15 or 30 days	Inhibition of liver mitochondrial lipid peroxidation, increased levels of reduced glutathione and enhanced activities of glutathione peroxidase, glutathione S-transferase, superoxide dismutase and catalase
Motawi T. M. K. et al., 2010 [32]	Male Wistar albino rats were administered for 7 days with SQ (0.4 mL/rat) before and after CP treatment	Improvement of GPx activity and GSH levels in cardiac tissue
Gabás-Rivera C. et al., 2014 [33]	Wild-type, Apoa1- and Apoe- deficient C57BL/6J male mice fed with 1 g/kg SQ for 11 weeks	Decreased ROS in lipoproteins
Ravi Kumar S. et al., 2016 [30]	Male KK-A^y^ mice fed high fat/sucrose diets supplemented with 2% SQ in combination with astaxanthin for 4 weeks	Elevated mRNA expression of SOD1 and GPx1 in liver

**Table 2:** Correlation or molecular mechanism-based studies showing the effect of SQ in different pathologies.

Pathological Condition		Reference	Effects
Positive effects of SQ	Hypercholesterolemia and atherosclerosis	Gabás-Rivera C. et al., 2014 [33]	Induction of paraoxonase-1 and reduction of ROS in lipoproteins in male mice fed with a SQ enriched diet
		Kritchevsky D. et al., 1954 [48]	Only correlation between anti-atherosclerotic effect and SQ enriched diet
		Guillén N. et al., 2007 [49]	Only correlation between reduced plasma cholesterol and triglycerides and SQ enriched diet in female mice ApoE-KO
		Hien H.T.M. et al., 2017 [50]	Activation of LXR α and β in macrophages
		Hoang T.M.H. et al., 2016 [51]	Activation of PPARα, induction of genes involved in lipid metabolism with hypolipidemic effect in HepG2 cells
		Granados-Principal S. et al., 2012 [52]	Reduced expression of CD36 in monocytes and macrophages
		Shin D.H. et al., 2004 [54]	Enhancement of cholesterol and biliary acid excretion in rats fed SQ enriched diet; inhibition of HMG-CoA reductase activity after i.p. injection of SQ in rats
		Hamadate N. et al., 2015 [76]	Enhancement of arterial elasticity in middle-aged and elderly men fed with shark liver enriched diet
	Hypertension	Martirosyan, D.M. et al., 2007 [56]	Reduction of systolic blood pressure after SQ-rich amaranth oil supplemented diets
		Liu Y. et al., 2009 [57]	Decrease of leptin levels and consequent reduction of plasma lipids and blood pressure
	Hyperglycemia-induced endothelial dysfunction	Liu Y. et al., 2009 [57]	Lower glycemia in rats fed SQ supplemented diet
Negative effects of SQ	Coronary Artery Disease	Rajaratnam R.A. et al., 2000 [63]	Only correlation between elevated plasma ratio of SQ to cholesterol and augmented risk for coronary artery disease in postmenopausal women
	Metabolic syndrome	Peltola P. et al., 2006 [65]	Only correlation between high serum SQ levels and visceral obesity
